# Glaucoma, Alzheimer's Disease, and Parkinson's Disease: An 8-Year Population-Based Follow-Up Study

**DOI:** 10.1371/journal.pone.0108938

**Published:** 2014-10-02

**Authors:** I-Chan Lin, Yuan-Hung Wang, Tsung-Jen Wang, I-Jong Wang, Yun-Den Shen, Nai-Fang Chi, Li-Nien Chien

**Affiliations:** 1 Department of Ophthalmology, Shuang Ho Hospital, Taipei Medical University, Taipei, Taiwan; 2 Division of Urology, Department of Surgery, Shuang Ho Hospital, Taipei Medical University, Taipei, Taiwan; 3 Graduate Institute of Clinical Medicine, College of Medicine, Taipei Medical University, Taipei, Taiwan; 4 Department of Ophthalmology, Taipei Medical University Hospital, Taipei Medical University, Taipei, Taiwan; 5 Department of Ophthalmology, National Taiwan University Hospital, School of Medicine, National Taiwan University, Taipei, Taiwan; 6 Department of Neurology, Shuang Ho Hospital, Taipei Medical University, Taipei, Taiwan; 7 Department of Neurology, School of Medicine, Taipei Medical University, Taipei, Taiwan; 8 School of Health Care Administration, College of Public Health and Nutrition, Taipei Medical University, Taipei, Taiwan; University of Melbourne, Australia

## Abstract

**Background:**

Glaucoma is the leading cause of irreversible blindness worldwide and primary open-angle glaucoma (POAG) is the most common type of glaucoma. An association between POAG and the subsequent risk of Alzheimer's disease (AD) and Parkinson's disease (PD) was unclear.

**Objective:**

To investigate the association between POAG (including normal-tension glaucoma) and the subsequent risk of AD or PD 8 years following a diagnosis of POAG.

**Methods:**

We performed a retrospective, propensity-score-matched analysis of a population-based cohort consisting of patients with and without POAG aged 60 years and older. Control patients without POAG were propensity-score matched to POAG patients based on their baseline characteristics.

**Results:**

The incidence rates and confidence intervals (CIs) of AD among the patients with and without POAG were 2.85 (95% CI: 2.19–3.70) and 1.98 (95% CI: 1.68–2.31) per 1000 person-years, respectively. The incidence rates of PD among the POAG and non-POAG cohorts were 4.36 (95% CI: 3.52–5.39) and 4.37 (95% CI: 3.92–4.86) per 1000 person-years, respectively. Kaplan-Meier failure curves showed that the POAG patients had a higher risk of AD than the control patients did (log-rank test, P = .0189). However, the cumulative PD hazard ratios for the POAG and non-POAG patients did not differ significantly (log-rank test, P = .9953).

**Conclusion:**

In elderly patients, POAG is a significant predictor of AD, but POAG is not a predictor of PD.

## Introduction

Glaucoma, a group of diseases characterized by progressive optic nerve degeneration, is the leading cause of irreversible blindness worldwide. Glaucoma affects more than 60 million people, and it has been estimated that the disease causes approximately 8 million people to develop bilateral blindness [1]. Primary open-angle glaucoma (POAG) is the most common type of glaucoma. The death of retinal ganglion cells (RGCs) is crucial to the pathophysiology of all forms of glaucoma. RGCs are a population of central nervous system (CNS) neurons with soma in the inner retina and axons in the optic nerve. Progressive loss of RGCs and atrophy of the optic nerve result in irreversible visual field (VF) loss.

Alzheimer disease's (AD) is a progressive neurodegenerative disorder characterized by cognitive and memory deterioration, changes in personality, behavioral disturbances, and an impaired ability to perform activities of daily living. As most common form of dementia, AD is a major public health problem worldwide. Glaucoma and AD share several features. Both are slow, chronic neurodegenerative disorders with an age-related incidence. Structural studies have shown that the optic nerves of both POAG and AD patients exhibit degeneration and loss of RGNs [2], [3]. On a molecular level, caspase activation induces abnormal amyloid precursor protein (APP) formation, which is the key event in the pathogenesis of AD, were observed in a rat model of chronic ocular hypertension [4]. Although several clinical studies have demonstrated an increased prevalence of POAG in AD patients [5], [6], large population-based studies have not revealed an association between POAG and AD [7], [8]. Therefore, the relationship between glaucoma and AD remains unclear.

Parkinson disease (PD) is a progressive neurodegenerative disorder characterized by the selective loss of dopaminergic neurons in the nigrostriatal pathway. Patients with PD exhibit slow movement, difficulty walking, rigidity, and tremor. The specific cause of PD is unknown. Small-sample retrospective studies have reported that PD patients are likely to exhibit glaucomatous-like VF defects [9], [10]. Studies using optic coherence tomography have shown that peripapillary retinal nerve fiber (RNFL) thinning occurs in PD [11]–[13]. However, no large population-based studies have demonstrated that POAG patients are likely to develop PD.

The purpose of our study is to investigate the association between POAG [including normal-tension glaucoma (NTG)] and the subsequent risk of AD or PD 8 years after POAG diagnosis. We hypothesized that POAG patients exhibit a higher risk of developing AD or PD, compared with the matched non-POAG controls.

## Methods

### Study design and data set

We used a retrospective population-based cohort design to perform a propensity-score-matched case-control analysis. The study cohort consisted of a group of patients diagnosed with POAG and a control group of patients who had not been diagnosed with POAG. The case and control patients were selected from the Taiwan National Health Insurance Research Database (NHIRD). The NHIRD was provided by the National Health Insurance Administration (NHIA) of Taiwan and contains claims data on inpatients and outpatients as well as the prescription records and demographic and enrollment profiles of beneficiaries. In this study, we used a data set of 2 million beneficiaries randomly sampled from the 23 million people in the general Taiwanese population. The data set is maintained by the Collaboration Center of Health Information Application (CCHIA), Ministry of Health and Welfare. This Study used NHIRD data collected from 2000 to 2009.

### Ethics Statement

According to CCHIA regulations, individual identifiers are encrypted to protect the privacy of beneficiaries and are released to investigators for research purposes. The data can be used only in an independent CCHIA operation zone and only statistical results can be brought out from the zone. This study was approved by the Institutional Review Board of Taipei Medical University (No. 201306031).

### Study patients

The POAG cohort comprised patients with at least 2 recorded visits for POAG (ICD-9-CM codes: 365.10 Open Angle Glaucoma, unspecified; 365.11 Primary Open Angle Glaucoma; 365.12 Normal Tension Glaucoma) between 2001 and 2008. 2 recorded visits should be with the same diagnosis code, and the 2 visits should be at least 30 days apart. In addition, we limited our study sample to those patients who had received antiglaucoma medication or had undergone glaucoma surgery during the study period. The first diagnostic date was used as the index date. Only those who had ever visited any ophthalmology clinic or department of ophthalmology of any hospital during study period were included. These cases of POAG were all diagnosed by certified ophthalmologists.

Patients with a history of AD (ICD-9-CM: 331.0), other forms of dementia (ICD-9-CM: 290, 290.0–4, and 290.8–9), or PD (ICD-9-CM: 332), or who had received a prescription of acetylcholinesterase inhibitors [AChEIs, Anatomical Therapeutic Chemical (ATC): N06DA02, N06DA03, N06DA04] or an anticholinergic agent (ATC: N04AA01, N04AA02) and dopaminergic agent (ATC: N04BA01, N04BA02, N04BB01, N04BC02, N04BC02, N04BC04, N04BC05, N04BC06, N04BC07, N04BD01, N04BX02) were excluded. We used a “look-back” period of at least one year to ensure that patients had not developed AD or PD before the index date. We selected only patients who were 60 years and older.

Control patients were selected from the remaining sample in the NHIRD, and consisted of patients with no diagnosis of POAG during 2001 to 2008. We randomly assigned a pseudodiagnosis date that corresponded to the index date of the pools of the POAG patients. The pseudodiagnosis date was used to ensure that the POAG patients and non-POAG patients were observed for the same length of time. We also ensured that the non-POAG patients had no history of AD or PD before the pseudodiagnosis date.

We used a 1∶4 case-control matching analysis based on propensity scores. The POAG patients were matched to controls according to the predicted probability of exposure, referred to here as a diagnosis of POAG. The matched cases and controls were similar for all covariates used to calculate the propensity score [14]–[16]. This method is commonly used in observational studies to reduce the sample selection bias [17]–[19].

### Study variables

The covariates that were used to calculate the propensity score were age, sex, previous or coexisting medical conditions, insurance eligible category, monthly income, urbanization level, and diagnostic year. The major disease conditions were hypertension (ICD-9-CM: 401–405), diabetes (ICD-9-CM: 250), ischemic heart disease (ICD-9-CM: 410), and stroke (ICD-9-CM: 430–438), which are known to associate with AD [20]–[21]. We also used the Charlson comorbidity index (CCI) as a summary score to represent the severity of disease conditions. The CCI consists of 22 disease conditions, and each condition is assigned a score of 1, 2, 3, or 6, depending on the risk of dying associated with each condition [22]. Health studies, including glaucoma studies [7], [23], have often used it to measure the disease burden and case mix [24], [25]. To increase the validity of disease diagnosis, patients with at least 2 health visits for a certain disease and 2 visits that were at least 4 weeks apart were considered as having a specific disease. Our study used monthly income for premium, eligible insurance categories, and urbanization level as proxy indicators to represent a patient's socioeconomic status, which has been shown to be associated with treatment for glaucoma [26]–[30]. According to NHIA rules, the 6 eligible insurance categories are based on the occupational statuses of enrollees. We used these categories to create 3 groups, Category 1 comprised civil servants, institutional workers, and enterprise, business, and industrial administration personnel; Category 2 comprised farmers, fishermen, vendors, and industrial laborers; and Category 3 consisted of all other occupations. The urbanization level was classified into 4 categories according to the population density, percentage of residents with a college education or higher, percentage of residents over 65 years of age, percentage of residents who are agricultural workers, and number of physicians per 100 000 people [31].

### Main outcome measure

The main outcomes were a diagnosis of AD and PD. Patients who developed AD and PD on different occasions during the observational period were included in the analyses. To increase the validity of the diagnoses, we defined the patients as either AD or PD patients if they had at least 2 visits within a 30-day-period with the same diagnosis code and were prescribed medications for treating AD or PD during the same period. The date of the first AD or PD diagnosis code was defined as the disease onset date.

### Statistical analysis

The POAG and control patients were followed from the index date until December 31, 2009 or death. The follow-up periods ranged from 1 to 8 years. Death records were obtained from the National Death Registry. Standardized differences were used to evalute the covariates used to predict the propensity scores of matched pairs. Cox proportional hazard regression analysis was used to compare the risk of AD or PD between patients with POAG and the non-POAG matched control patients. The models were also passed through the proportional hazard assumption among cases and controls. SAS-STAT software (Version 9.3, SAS Institute, Cary, NC, USA) and STATA 12 software (Stata Corp, College Station, TX, USA) were used to perform all of the statistical analyses. A *P* value <.05 was considered to indicate statistically significant result.

## Results

The baseline characteristics after propensity score matching was conducted (Table 1) indicated that 52.9% of the patients in the study cohort were male, and the mean age for the study cohort was 71.3 years (Table 1). The non-POAG group included more patients aged 80 years and older (15.0%) than the POAG group did. The prevalence of previous and coexisiting medical conditions among the POAG cases was 74.1% for hypertension, 41.3% for diabetes, 0.3% for heart failure, and 26.3% for stroke. The CCI scores were distributed as follows: 10% CCI  = 0, 14.5% CCI  = 1, and 75.5% CCI ≥2. As shown in Table 1,the propensity score matching method distributed the case and matched controls fairly equally among all covariates.

**Table 1:** Demographic characteristics and comorbidities in cohorts with and without POAG.

Variable	POAG		P-value
	No	Yes	
	N = 15916	N = 3979	
**Sex**	n (%)	n (%)	
Male	8405(52.8)	2104(52.9)	0.938
Female	7511(47.2)	1875(47.1)	
Age, mean ± SD	71.3±7.41	71.3±7.08	0.791
**Stratify aAge**			0.099
60–69	6818 (42.8)	1701(42.7)	
70–79	6709(42.2)	1730(43.5)	
> = 80	2389 (15.0)	548(13.8)	
**Comorbidity**			
Hypertension	11858(74.5)	2950(74.1)	0.637
Diabetes	6572(41.3)	1645(41.3)	0.954
Heart failure	31(0.2)	12(0.3)	0.194
Stroke	4084(25.7)	1047(26.3)	0.399
**CCI score**			0.986
0	1605(10.1)	399(10.0)	
1	2311(14.5)	575(14.5)	
> = 2	12000(75.4)	3005(75.5)	
**Urbanization level**			0.971
1 (most urbanized)	9732 (61.1)	2429(61.0)	
2	4958(31.2)	1239(31.1)	
3 (least urbanized)	1226(7.7)	311(7.8)	

CCI  =  Charlson comorbidities index.

P-value: testing hypothesis of no difference between patients with and without POAG.

**Figure 1:**
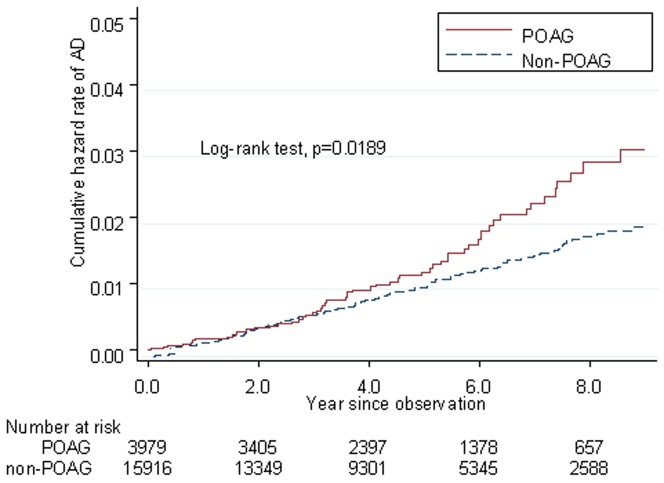
Kaplan-Meier failure curve of patients with POAG and non-POAG who developed AD after accounting for censoring due to death or end of observational period.

**Figure 2:**
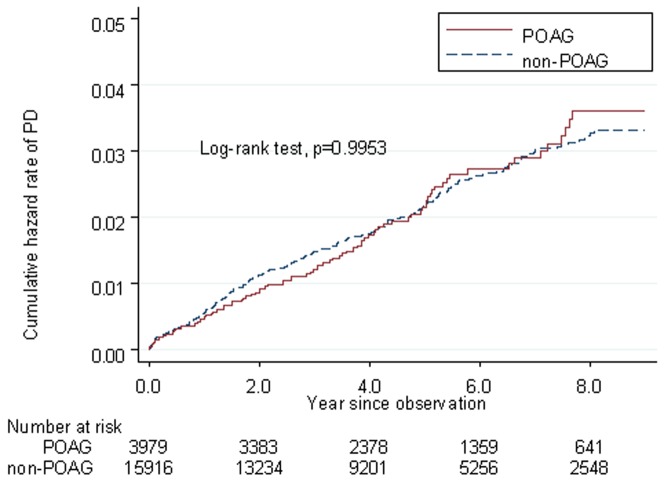
Kaplan-Meier failure curve of patients with POAG and non-POAG who developed PD after accounting for censoring due to death or end of observational period.

The incidence rates per 1000 person-years of AD among patients with POAG and the non-POAG controls were 2.85 (95% confidence interval [CI], 2.19–3.70) and 1.97 (95% CI, 1.68–2.31; Table 2), respectively. The incidence rates of PD among patients with and without POAG were 4.36 (95% CI, 3.52–5.39) and 4.37(95% CI, 3.92–4.86) per 1000 person-years, respectively. The Kaplan-Meier failure curves showed that the risk of AD among the patients with POAG was higher than that of the matched control patients (log-rank test, *P* = .0189, Figure 1); however, no significant difference (log-rank test, *P* = .9953) in the cumulative PD hazard ratio (HR) was observed between the POAG and control groups (Figure 2).

**Table 2:** The incidence rate per 1000 person-years of AD and PD among patients with and without POAG.

Outcome	PY	Failures (n)	Rate (95% CI)	Crude HR	Adjusted HR
AD					
POAG	19653	56	2.85 (2.19–3.70)	1.44 (1.06–1.96)	1.40(1.03–1.90)*
Non-POAG	76994	152	1.97 (1.68–2.31)		
PD					
POAG	19500	85	4.36 (3.52–5.39)	1.00 (0.97–1.27)	0.98(0.77–1.24)
NON-POAG	76282	333	4.37(3.92–4.86)		

POAG  =  Open angle glaucoma, AD  =  Alzheimer disease; CI  =  confidence interval; PD  =  Parkinson disease, HR  =  hazard ratio; PD  =  Parkinson disease.

Adjusted HR for age, sex, comorbidites (hypertension, diabetes, heart faiulre, stroke), insurance eligibility group, monthly income, diagnostic year, urbanization level and Charlson comorbidities index; score.

*P<.05.

The adjusted HR of AD in the POAG patients, relative to that of their matched controls, was 1.40(95% CI, 1.03–1.90, *P* = .033). The adjusted HR of PD in the POAG patients, relative to that of their matched controls, was 0.98 (95% CI, 0.77–1.24, *P* = .861). These data indicated that POAG increased the risk of AD, but did not increase the risk of PD. In 3979 POAG patients, 546 patients (13.7%) of them were diagnosed with NTG. Compared the NTG patients with their matched patients, the adjusted HR of AD was 1.39 (*P = .424*) and the adjusted HR of PD was 1.32 (*P = .329*).

## Discussion

The results of our population-based propensity-score-matched cohort study suggested that a clinical diagnosis of POAG increases the risk of developing AD, but not the risk for developing PD. The propensity score matching technique enabled us to identify comparable matched pairs; thus, patients with POAG and non-POAG matched control patients exhibited similar baseline characteristics. Using this approach prevents the selection bias that occurs in most observational studies and can substantially improve the validity of study results [32], [33].

Previous studies have indicated that the incidence of glaucoma among patients with AD is higher than that of patients without AD [5], [6], [34], [35]. Pseudoexfoliation syndrome (PEX) is the most important independent risk factor of open angle glaucoma (OAG), and the deposition of amyloid-like material in PEX shares some features with the findings in AD [36]. Cumurcu et al reported increased prevalence of AD in patients with PEX comparing to the control groups [34]. They concluded the PEX material deposition in anterior chamber could be a useful finding in early diagnosis of AD [34]. Ekstrom et al included a cohort including 1123 participants of aged 65–74 years in central Sweden [37]. At the baseline, 246 patients with PEX and 122 patients with newly diagnosed OAG were included.However, they did not find any significant association between PEX and AD or between OAG and AD over 30 years of follow-up [37].

Our findings were consistent with the result of the Three-City-Bordeaux-Alienor study that included a cohort of 812 participants who received eye and neuropsychological examination at the start and the end of a 3-year period [35], and it revealed a 3.9-fold increase in the risk of AD in POAG patients; however, the number of participants who developed dementia over the 3-year study period was relatively low, thus resulting in a wide confidence interval of relative risk (1.5–10.4). In this current study, the accuracy of claims data substantially influences the findings. Thus, we combined the diagnostic codes and the concurrent medication to increase the accuracy of the diagnosis. Additionally, a relative large sample size and a long follow-up period would also improve the risk estimation of AD in POAG (adjusted HR:1.39 with confidence interval  = 1.03–1.87). It is possible that patients with dementia might be misclassified as non-demented patients if they did not seek medical attention. However, the misclassification should be non-differential between POAG patients and non-POAG patients that might result in an underestimation of the hypothesized association between POAG and AD.

Two large population-based studies have investigated the relationship between glaucoma and AD [7], [8]. Kessing et al. used a national Danish case register of hospital admission data and subsequent outpatient visits to identify the AD diagnosis rates in the following cohorts: people with POAG, angle-closure glaucoma, cataracts, and osteoarthritis, and the general population [8]. The risk of developing AD was the same in the POAG cohort and the other cohorts (including the general population) [8]. However, the data contains the records of only inpatients but not outpatients before 1995, and the control group did not match the POAG cohorts according to demographics or a comorbidity index such as the CCI; therefore, bias and confounding factors could affect the results.

Ou et al. examined Medicare claims data and did not observe an association between AD and POAG [7]. One notable difference between our study design and that of Ou et al is that their cohorts consisted exclusively of patients aged 68 years or older. In western countries, AD affects 1% to 3% of people between the ages of 60 and 70 years [38]. Therefore, compared with our cohort, the study design used by Ou et al was more likely to exclude patients who had developed both AD and POAG before the age of 65 years.

Another difference between our study design and that used by Ou et al is that we compared the HR of AD between patients with NTG and their matched controls. Several studies had reported NTG was associated with AD and indicated this neurodegenerative aspect may be specific to NTG [5], [6]. However, a cohort study conducted by Bach-Holm et al showed no patients developed AD in 69 patients with NTG and the mean follow-up period was 12.7 years [39]. In this claim-based study, we found a positive but left in insignificant association between NTG and the subsequent development of AD (Adjusted HR  = 1.32, *P = .329*). NTG commonly is defined as POAG with untreated intraocular pressure of less than 22 mmHg, and NTG is also the most common form of POAG [40]. A previous study showed that NTG comprises the majority (52–92%) of POAG in Asian epidemiologic studies, which is much higher than that reported in a Caucasian population [41]. However, the proportion of NTG in POAG patients in our study is 13.7%, which is relatively lower than that reported in Asian epidemiologic studies.^41^ Due to NTG is defined as a subtype of POAG, some patients with NTG might be coded as unspecified POAG. Our databases did not provide information on intraocular pressure, and this limitation is likely to result in underestimation of the prevalence of NTG. Therefore, this study was unable to examine the association between NTG and AD.

Some studies with small sample sizes have indicated that PD patients are more likely to develop glaucoma-like VF defects [9], [10], [42]. Several studies have observed reduced RNFL thickness in PD patients [11], [12]. However, no published studies have proved that these VF defects in PD patients are correlated to RNFL thinning. In addition, all of these studies used a cross-sectional design and did not observe a causal relationship between POAG and PD. Our study is the first large population-based study that demonstrates that patients with POAG are unlikely to develop PD.

The large, nationally representative sample of elderly people used in our study lends credibility to the findings of our longitudinal analysis of AD and PD in POAG patients in Taiwan. However, our findings are nonetheless subject to certain limitations. First, the diagnoses of POAG, AD, PD, and comorbid medical conditions were based entirely on the ICD-9-CM codes used in the NHI claims data, and thus might be less accurate than diagnoses obtained through a standardized procedure. Second, the NHIRD did not provide information on glaucomatous optic neuropathy and VF defects. To ensure the validity of disease diagnoses, we assigned diagnoses based on claims for 2 visits at least 30 days apart with the same diagnosis code, as well as related prescription claims. This combined approach helped us to accurately identify patients with a specific disease. In addition, the NHIA routinely samples the claims data, and reviews the full medical records of beneficiaries to verify the accuracy of diagnoses. Hospitals and clinics are penalized if they have provided unnecessary medical treatment to misdiagnosed patients [43]. This practice of the NHIA suggests that our results are valid and robust.

The NHI claims data do not include information on blood pressure, blood glucose levels, or behavioral risk factors, such as diet, lifestyle, and cigarette smoking. Thus, simply including hypertension and diabetes in our covariate models might not have adequately adjusted for the confounding effects of blood pressure and blood glucose levels. Data on family history and genetics, both of which might be risk factors for AD and PD, are not included in the NHI claims data. These factors might have compromised our findings.

Undiagnosed POAG patients might also represent another limitation to our findings. Most studies have concluded that approximately 50% of glaucoma cases go undetected [44]. Recent studies have examined the epidemiology of glaucoma in Asian people and reported that the prevalence of glaucoma range from 2.1% to 5% [45]. We can infer from these data that approximately 1.05% to 2.5% of the population has undetected glaucoma. These patients with undetected glaucoma are categorized as nonglaucoma patients according to the criteria used in this study and have a small chance of being selected as part of the comparison cohort. However, the inclusion of patients with undetected glaucoma in our comparison cohort would have resulted in an underestimated risk. Therefore, our observation of an increased incidence of AD in patients with POAG likely reflects an actual increase in the risk of AD. If we want to ensure the subclinical POAG to be excluded in the control group, a prospective study design with a specialist's evaluation before each subject enrollment is needed.

In longitudinal studies of frail populations, such as elderly people, death can occur prior to the occurrence of the disease-related outcome measurement [46]. Therefore, standard survival predictions for such populations can substantially overestimate the absolute risk of the outcome event because participants with a competing event are treated (and thus censored) as if they can experience the outcome event in the future. Thus, a completive risk model should be considered for follow-up studies in elderly populations. Fortunately, we had a consistent finding in both models and a competitive risk model resulted in a 1.40 times increased risk of AD (*P = .03*).

Surveillance bias might also have occurred because patients with POAG might be more likely to visit clinics than patients without POAG are. However, we used propensity scores to balance the incidences of major underlying diseases between the POAG and control patients. Therefore, the need for medical attention was likely similar between the POAG and control patients, resulting in a similar likelihood of having AD detected. The impact of surveillance bias on the risk of AD in POAG patients was likely minimal. However, the surveillance bias could still be a factor affected the risk of PD and AD.

In conclusion, our population-based study demonstrated that POAG is a significant predictor for the development of AD, but POAG is not a predictor of PD. Future studies are warranted to confirm our results, and identify the underlying pathological mechanism that contributes to the development of AD in POAG patients.
