# Correction: Glaucoma, Alzheimer's Disease, and Parkinson's Disease: An 8-Year Population-Based Follow-Up Study

**DOI:** 10.1371/journal.pone.0150789

**Published:** 2016-03-01

**Authors:** I-Chan Lin, Yuan-Hung Wang, Tsung-Jen Wang, I-Jong Wang, Yun-Dun Shen, Nai-Fang Chi, Li-Nien Chien

The fifth author’s name is spelled incorrectly. The correct name is: Yun-Dun Shen.
